# Cervical Intradural Disc Herniation Causing Progressive Quadriparesis After Spinal Manipulation Therapy: A Case Report and Literature Review

**DOI:** 10.1097/MD.0000000000002797

**Published:** 2016-02-12

**Authors:** Hwan-Seo Yang, Young-Min Oh, Jong-Pil Eun

**Affiliations:** From the Department of Neurosurgery, Biomedical Research Institute, Chonbuk National University Medical School and Hospital, Jeonju, Korea.

## Abstract

Cervical intradural disc herniation (IDH) is an extremely rare condition, comprising only 0.27% of all disc herniations. Three percent of IDHs occur in the cervical, 5% in the thoracic, and over 92% in the lumbar spinal canal. There have been a total of 31 cervical IDHs reported in the literature. The pathogenesis and imaging characteristics of IDH are not fully understood. A preoperative diagnosis is key to facilitating prompt intradural exploration in patients with ambivalent findings, as well as in preventing reoperation. The purpose of reporting our case is to remind clinicians to consider the possibility of cervical IDH during spinal manipulation therapy in patient with chronic neck pain.

The patient signed informed consent for publication of this case report and any accompanying image. The ethical approval of this study was waived by the ethics committee of Chonbuk National University Hospital, because this study was case report and the number of patients was <3.

A 32-year-old man was transferred our emergency department with progressive quadriparesis. He had no history of trauma, but had received physical therapy with spinal manipulation for chronic neck pain over the course of a month. The day prior, he had noticed neck pain and tingling in the bilateral upper and lower extremities during the manipulation procedure. The following day, he presented with bilateral weakness of all 4 extremities, which rendered him unable to walk. Neurological examination demonstrated a positive Hoffmann sign and ankle clonus bilaterally, hypoesthesia below the C5 dermatome, 3/5 strength in the bilateral upper extremities, and 2/5 strength in the lower extremities. This motor weakness was progressive, and he further complained of voiding difficulty.

Urgent magnetic resonance imaging (MRI) of the cervical spine revealed large, central disc herniations at C4–C5 and C5–C6 that caused severe spinal cord compression and surrounding edema. We performed C4–C5–C6 anterior cervical discectomy and fusion.

The patient's limb weakness improved rapidly within 1 day postoperatively, and he was discharged 4 weeks later. At his 12-month follow-up, the patient had recovered nearly full muscle power.

We presented an extremely rare case of cervical IDH causing progressive quadriparesis after excessive spinal manipulation therapy. The presence of a “halo” and “Y-sign” were useful MRI markers for cervical IDH in this case.

## INTRODUCTION

Intradural disc herniation (IDH) is an extremely rare condition, comprising only 0.27% of all disc herniations and there have been a total of 31 cervical IDHs reported in the literature.^1–4^ Because the pathogenesis and imaging characteristics of IDH are not fully understood, a preoperative diagnosis is key to facilitating prompt intradural exploration in patients with ambivalent findings, as well as in preventing reoperation.^5^ We provide 1 additional case report, along with a brief review of the literature.

## CASE REPORT

A 32-year-old man was transferred our emergency department with rapidly progressive quadriparesis. He had no history of trauma, but had received physical therapy with spinal manipulation for chronic neck pain over the course of a month. The day prior, he had noticed neck pain and tingling in the bilateral upper and lower extremities during the manipulation procedure. The following day, he presented with bilateral weakness of all 4 extremities, which rendered him unable to walk. Neurological examination demonstrated a positive Hoffmann sign and ankle clonus bilaterally at that time, although these reflexes were not checked before. He complained hypoesthesia below the C5 dermatome, 3/5 strength in the bilateral upper extremities, and 2/5 strength in the lower extremities. This motor weakness was progressive, and he further complained of voiding difficulty.

**FIGURE 1:**
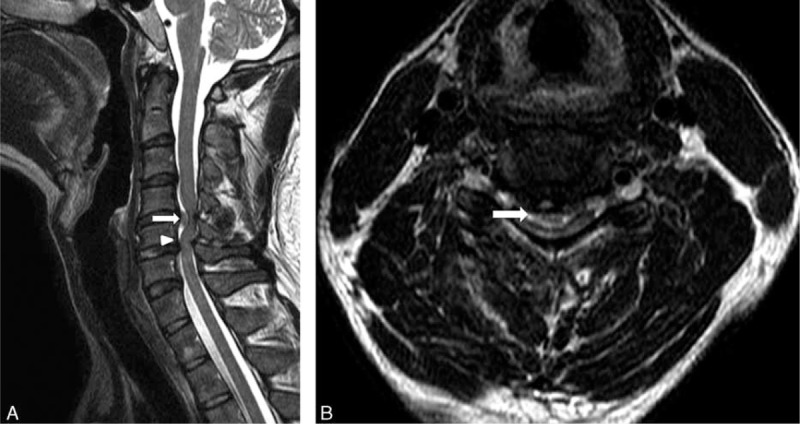
Preoperative MRI. (A) Sagittal T2-weighted image of the cervical spine revealed large disc herniation at C4–C5 (arrow) and C5–C6 with severe spinal cord compression. The “Y-sign” (arrow head) was found. (B) Axial T2-weighted image of the cervical spine revealed large, central disc herniation at C4–C5 with severe spinal cord compression and surrounding edema. A “halo” of CSF isointensity surrounding the herniated disc was also demonstrated (arrow). CSF = cerebrospinal fluid, MRI = magnetic resonance imaging.

**FIGURE 2:**
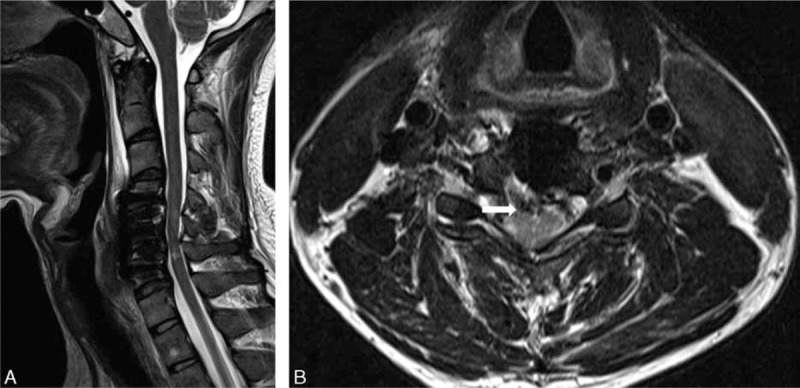
MRI taken 3 weeks postoperatively showed high signal intensity in the spinal cord at C4–C5 and C5–C6 (A), as well as adhesions of the spinal cord to the PLL (arrow) (B). MRI = magnetic resonance imaging, PLL = posterior longitudinal ligament.

Urgent magnetic resonance imaging (MRI) of the cervical spine revealed large, central disc herniations at C4–C5 and C5–C6 with severe spinal cord compression and surrounding edema (Figure 1). Furthermore, an area of increased intensity was seen in the spinal cord at C5–C6. The patient underwent anterior cervical discectomy and fusion at the C4/5/6 level. A large sequestrated disc had perforated through the posterior longitudinal ligament (PLL) into the intradural space, with an adhesion to the dura and PLL. We removed the extruded disc material, along with a small, longitudinal defect in the PLL and dura, under microscopic guidance. The dural tear was closed with Tachocomb, and an interbody cage filled with allograft and an anterior cervical plate was placed at C4/5/6. The patient's limb weakness improved rapidly within 1 day postoperatively, and he was discharged 4 weeks later. MRI taken 3 weeks postoperatively showed high signal intensity in the spinal cord at C4–C5 and C5–C6, as well as adhesions of the spinal cord to the PLL (Figure 2). At his 12-month follow-up, the patient had recovered nearly full muscle power.

## DISCUSSION

Since Marega^6^ reported the first case of intradural cervical disc rupture, 31 cases of this rare condition have since occurred in the literature. In our review of these 32 cases, including our new case, we found a male predominance (19 of 32 cases) in IDH incidence, and the mean age at presentation was 45.2 years. The lower cervical spine, a site of increased spinal mobility, was the most frequently affected, particularly at C5–C6 and C6–C7.^2^ The most common clinical manifestations of cervical IDH were Brown–Sequard syndrome, transverse myelopathy, and rarely radiculopathy.^4^ In our new case, the patient presented with rapidly progressive quadriparesis after spinal manipulation therapy.

### Pathogenesis of Cervical IDH

The mechanism of cervical IDH remains unclear. Trauma has been considered the most important inciting factor in previous studies,^2,7^ suggesting that traumatic injury may cause chronic adhesions between the ligaments and dura; indeed, these adhesions were found intraoperatively in most cervical IDH cases. In our patient, disc protrusion and repeated spinal manipulation caused chronic inflammation and mechanical irritation of the adjacent dura, creating adhesions between the PLL and the dura. We posit that the spinal manipulation procedures caused transdural disc herniation that compressed the spinal cord. Furthermore, we found evidence of adhesions between the dura and PLL in our patient intraoperatively.

Yildizhan et al^8^ performed a cadaveric study that found the dura to be firmly attached to the PLL in some cases. They hypothesized that congenital factors may have a role in the pathogenesis of cervical disc herniation.

Some authors have reported cases of cervical IDH in patients with ossification of PLL.^9,10^ They suggested that PLL hypertrophy and ossification can cause chronic mechanical irritation to the dura, particularly if it is already congenitally attached to the ligament.

### MRI Findings in Cervical IDH

Because it is difficult to identify dural sac perforation on MRI, there have been few reports on the imaging features of IDH. In 1998, Hidalgo-Ovejero et al^11^ suggested that the presence of epidural gas on computed tomography could be an indicator. Choi et al^5^ designated an abrupt discontinuity in the PLL “hawk-beak sign” on MRI. Recently, Borm et al^12^ described a “Halo” of cerebrospinal fluid (CSF) isointensity surrounding a herniated mass on sagittal T2-weighted imaging that could indicate intradural extension into the cervical spine. Similarly, Sasaji et al^13^ described a “Y-sign” in the lumbar spine. Because intradural extra-arachnoid disc herniation occurs between the dura and arachnoid, the arachnoid was peeled from the dura by the disc herniation. One line of the dura and arachnoid was divided into 2 lines of the dura and the arachnoid. The branch of the ventral dural lines appeared as “Y,” and they called it the “Y sign.” In our case, a “halo” of CSF isointensity surrounding the herniated disc was demonstrated in axial MR images (Figure 1B), and the “Y-sign” was found in sagittal MR images (Figure 1A). We found that the CSF isointensity around the herniated area appeared not only as a “halo” in axial images, but also as “Y-sign” on sagittal images. We therefore suggest that both the “halo” and “Y-sign” are strong indicators of IDH on MRI.

### Surgical Treatment of Cervical IDH

Surgical intervention is the definitive treatment for cervical IDH, and all patients in the literature underwent surgery. According to previous reports, anterior cervical discectomy and fusion is superior to posterior decompression in terms of operative procedure and outcome.^1,10,14^ Since the anterior approach directly accesses the affected disc, it facilitates removal of extruded disc material and dura repair.

## CONCLUSION

We presented an extremely rare case of cervical IDH causing progressive quadriparesis after excessive spinal manipulation therapy. The presence of a “halo” and “Y-sign” were useful MRI markers for cervical IDH in this case.
